# Coupling reconstruction of atmospheric hydrological profile and dry-up risk prediction in a typical lake basin in arid area of China

**DOI:** 10.1038/s41598-022-10284-y

**Published:** 2022-04-20

**Authors:** Jie Wang, Dongwei Liu, Songni Tian, Jiali Ma, Lixin Wang

**Affiliations:** 1 School of Ecology and Environment, Inner Mongolia University, Hohhot, 010021 China; 2 Inner Mongolia Key Laboratory of River and Lake Ecology, Hohhot, 010021 China; 3 Key Laboratory of Ecology and Resource Use of the Mongolian Plateau, Ministry of Education of China, Hohhot, 010021 China

**Keywords:** Hydrology, Wetlands ecology, Environmental impact, Climate and Earth system modelling, Climate-change impacts

## Abstract

Arid area is very sensitive to global warming and are extremely vulnerable to climate change. Moreover, the water resources system in the arid area is fragile and will undergo tremendous changes with climate change. Therefore, the interaction of climate and hydrology in arid area has an important impact on the formation of regional microclimate and hydrological changes. Daihai Lake is a typical closed inland lake in arid area of China, and a key area for ecological protection in North China. In this paper, WRF-Hydro model is used to simulate the climate hydrological coupling situation of Daihai Basin from 1980 to 2020, and the coupling results are verified and calibrated by meteorological statistics, runoff calculation and remote sensing analysis. Based on the synopsis of climate and hydrology in the past 40 years, the causes and future trends of the hydrological elements in Daihai Basin are analyzed. Through the analysis, it is found that the interannual variation of precipitation in Daihai Basin is sharp, with 401.75 mm as the average from 1980 to 1994; and drastic fluctuations from 1995 to 2011, with a difference of nearly 400 mm between the interannual maximum and minimum; From 2012 to 2020, the fluctuation is small. Although the interannual variation of evaporation fluctuated, it showed an upward trend with a slope of 8.855 mm/year. The annual average temperature showed an obvious upward trend with a slope of 0.040 °C/year. From 1980 to 2020, the inflow of Daihai Lake shows a downward trend; Since 2013, the runoff into the lake has tended to be flat. Climate change and human activities are the decisive factors leading to the change of water quantity in Daihai, among which human activities play a greater role. Cultivated land irrigation and industrial water use are highly correlated with the lake discharge, and these two factors have a great influence on the lake discharge. If the current agricultural and industrial water consumption does not increase, Daihai still has a lifespan of nearly 120 years. If human activities do not change and any protective measures are not taken in time, under the background of global climate change, the flow of the Daihai Lake into the lake will be reduced to zero in 2025, and the Daihai Lake will completely dry up in 2031–2033. The study of climate hydrological coupling of long time series in Daihai Basin can not only make up for the lack of runoff data, but also provide the basis for water resources management, disaster prevention and mitigation.

## Introduction

The arid area has a fragile ecological environment and is extremely sensitive to climate change and human activities^1,2^. In particular, the weather, regional microclimate and hydrological processes in arid areas are extremely vulnerable^2,3^. With the warming of the global climate and the enhancement of high-intensity human activities, the local water cycle process in arid areas may have changed, resulting in frequent floods, droughts, blizzards and other disasters, and the ecological environment and human life have also been severely affected^4^. In recent years, the impact of climate change on hydrological events in arid areas has also been widely concerned^5^. However, there is a lack of comprehensive mechanism to explain how climate affects hydrological processes and how hydrological processes feedback to weather and climate. Therefore, it is particularly important to consider the coupling process of atmospheric hydrology in hydrological simulation in arid areas with variable hydrographic topography.

Hydrological cycle is an important part of the climate system. Climate change will certainly cause the change of hydrological information (water level, flow, groundwater level, etc.) in time and space^6,7^. For the basin in arid and semi-arid areas, the small change of climate elements in long time series will also cause the fluctuation of basin information sensitivity^8^. Under the background of fragile water resources system in arid area, the fluctuation and uncertainty of hydrological information will also have a negative impact on the microclimate. Zhang et al. found that in response to climate change, lakes on the Qinghai-Tibet Plateau gradually expanded, and lakes on the Mongolian Plateau gradually shrank. This shows that although the global climate is generally warming, the interaction between the regional microclimate and the water cycle is also very complex^7^. The study of climate hydrological coupling model in arid area can not only obtain the change of hydrological information under the background of climate change, but also obtain the impact of hydrological information change on regional microclimate. Scholars at home and abroad have done a lot of research in this area. Liu et al. Coupled GCM with Mike she to simulate the water resources change in different climate change scenarios in the Karakoram region in different future periods (2020–2039, 2040–2059, 2060–2079, 2080–2099). They found that in the case of climate change, the increase of evapotranspiration will lead to the decrease of snow cover in high-altitude mountainous areas and the decrease of available water in the downstream areas, At the same time, they also found that in the future climate scenario, the conversion between different forms of water resources will change the distribution of hydrological components^9^. He et al. integrated the meteorological model MM5 and hydrological model SWAT to couple hydrological dynamics with climate change in the Qinling Mountains to reveal the impact of climate on hydrological conditions in the context of global change from 1950 to 2005. They found that climate change is the cause of coupling effect changes of precipitation, land use, river flow and water resources. At the same time, it is found that the climate has become drier and the hydrological process has changed greatly in the past few decades^10^. Qin et al. used five climate models in CMIP5 coupled with three RCPs rain SWAT models to predict the impact of climate change on the hydrological information distribution in the upper reaches of the Yangtze River in the twenty-first century. The results showed that the temperature and precipitation in the upper reaches of the Yangtze River showed an increasing trend. After the 1930s, the average climate value was higher than that in the early twenty-first century, and the annual runoff also showed an increasing trend. The frequency and intensity of extreme drought and flood also increased significantly^11^.

At present, there are two coupling modes of climate and hydrology: one-way coupling and two-way coupling. Unidirectional coupling is a one-way forcing of climate models that drives hydrological models and does not accept feedback from hydrological models. Bidirectional coupling means that after the climate model drives the hydrological model, it also accepts the feedback from the hydrological model and adjusts the output results^12^. The focus of unidirectional coupling research is the influence of climate model accuracy on runoff prediction, which lacks the overall description of water cycle. Meanwhile, climate models cannot accept real-time feedback from hydrological models to verify and improve the description of climate components^13^. For arid areas, due to the lack of meteorological and hydrological stations, the accuracy of meteorological models is low. The feedback of hydrological models to meteorological models can improve the accuracy and description power of the output components of meteorological models. Meanwhile, two-way coupling can also bring the high resolution of hydrological models into the climate models, so as to obtain a more detailed description of the regional environment. Amir et al. calibrated and evaluated the hydroclimate model system for the Ayalon Basin in central Israel using WRF, WRF-Hydro, and HEC-HMS to evaluate the advantages and disadvantages of one-way and two-way coupling. They found that using two-way coupling can improve the simulation of precipitation for water-deficient areas and provide more accurate flood information, but WRF simulations are much better than WRF/WRF-Hydro simulations for winter precipitation^14^. In addition, Senatore et al. found that WRF/WRF-Hydro bi-directional coupling was more effective in improving the simulation of precipitation, runoff and other elements in inland areas where soil moisture caused severe convective weather and in areas with complex terrain and relatively dry areas with obvious lateral distribution of water^15^. Silver et al. used WRF/ WRF-Hydro two-way coupling to conduct runoff simulation in arid and semi-arid areas of Jordan and Israel, and found that the predicted value could truly obtain runoff conditions and variation rules^16^. The two-way coupling can not only improve the simulation accuracy of climate and hydrological factors, but also obtain the influence of hydrological information changes on climate. Zhang et al. used WRF/WRF-Hydro to compare the fitting results from 2008 to 2010 to evaluate the influence of horizontal surface flow on the interaction of surface atmosphere. They found that horizontal surface flow could increase evapotranspiration and reduce total runoff, and the resulting increase in near-surface humidity and decrease in temperature could affect climate through water vapor content^17^. WRF-Hydro model, as a two-way coupling model of atmospheric and hydrology, has been increasingly applied in the field of meteorology and hydrology. WRF-Hydro is the hydrological extension of WRF model^14^. Compared with WRF model, WRF-Hydro can simulate hydrological processes on land surface at a higher spatial scale. In addition, the simulation of land surface heat and water flux in WRF model is improved, so as to finally improve the simulation of precipitation and land water cycle in WRF model^17,18^.

In this study, Daihai Basin is taken as a typical lake basin in arid area of China. WRF-Hydro model is used to simulate the climate hydrological coupling in Daihai Basin. Meteorological statistics, runoff generation calculation and remote sensing analysis are used to verify and calibrate the coupling results. This paper reconstructs the process of climate and runoff changes in the Daihai Basin from 1980 to 2020, and analyzes the causes and future trends of the changes in hydrological elements in the Daihai Basin. This provides an important foundation for water resources management, disaster prevention and mitigation, and water ecological environmental protection in the Daihai Basin.

## Materials and methods

### Study area

**Figure 1:**
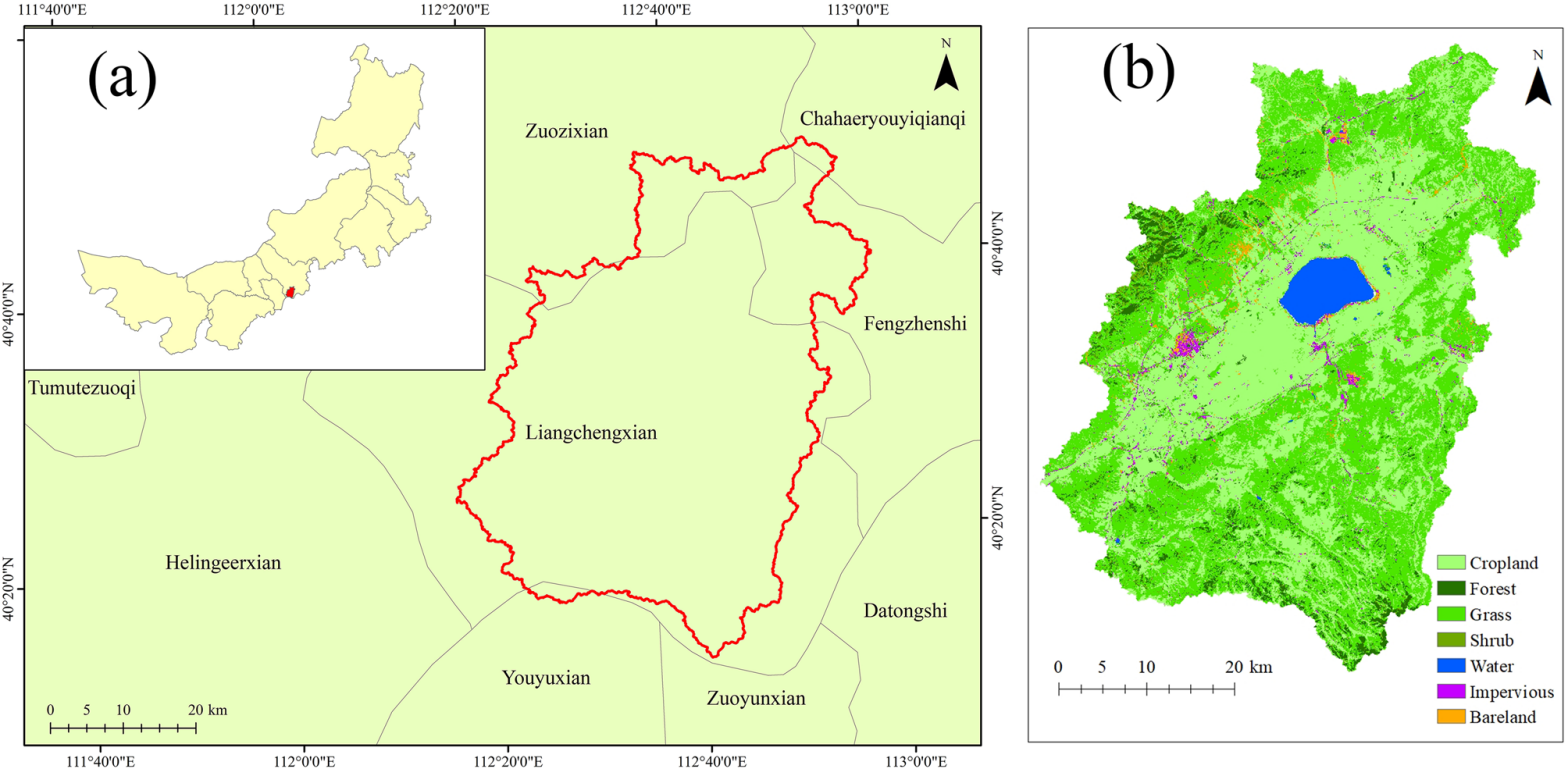
Overview of the study area: (**a**) Geographical location of the Daihai Basin; (**b**) Land Use in the Daihai Basin.

Daihai Basin is a typical inland closed lake basin, which is located in the central part of Inner Mongolia. It includes the Liangcheng County, Fengzhen City and Zhuozi County in Ulanqab City, Inner Mongolia^19^. Daihai Basin is located in 112°10'E ~ 112° 59'E, 40° 48'N ~ 40° 55'N, with an area of 2312.75 km^2^ (Fig. 1, produced by ArcGIS10.6, https://desktop.arcgis.com/de/arcmap/10.6/, the administrative boundary data comes from the Resource and Environmental Science and Data Center of the Chinese Academy of Sciences, and the land use data comes from the MODIS MOD12Q1 product). Daihai Basin is surrounded by mountains, among which the highest peak Manhan Mountain (Yinshan branch) is 2305 m and the lowest is 1218 m located at Daihai Lake^20^. Daihai Basin belongs to temperate continental climate, with long cold winter and short high heat summer. The annual average temperature of Daihai Basin is 5.0 °C, the extreme maximum temperature over the years is 39.3 °C (July 7, 2005)^21^, and the minimum temperature over the years is − 34.5 °C. The average annual precipitation is 403.98 mm, which is unevenly distributed during the year, mostly from June to September. The annual average evaporation is 1030.11 mm. The average annual wind speed is 2.4 m/s. There are 22 rivers flowing into Daihai Basin, most of which are intermittent rivers and only have runoff in rainy season.

### Basic methods

#### Numerical simulation

WRF-Hydro model is used to simulate the climate and hydrology of Daihai Basin. WRF-Hydro provides a method to couple hydrological model with atmospheric model and other earth system models. Compared with WRF, WRF-Hydro improves the hydrological process expression of spatial redistribution of surface water, groundwater and river water in the model, and promotes the coupling of hydrological model and atmospheric model^22^. Different land hydrological physical models can be selected in WRF-Hydro, which is created or improved from the existing distributed hydrological models.

**Figure 2:**
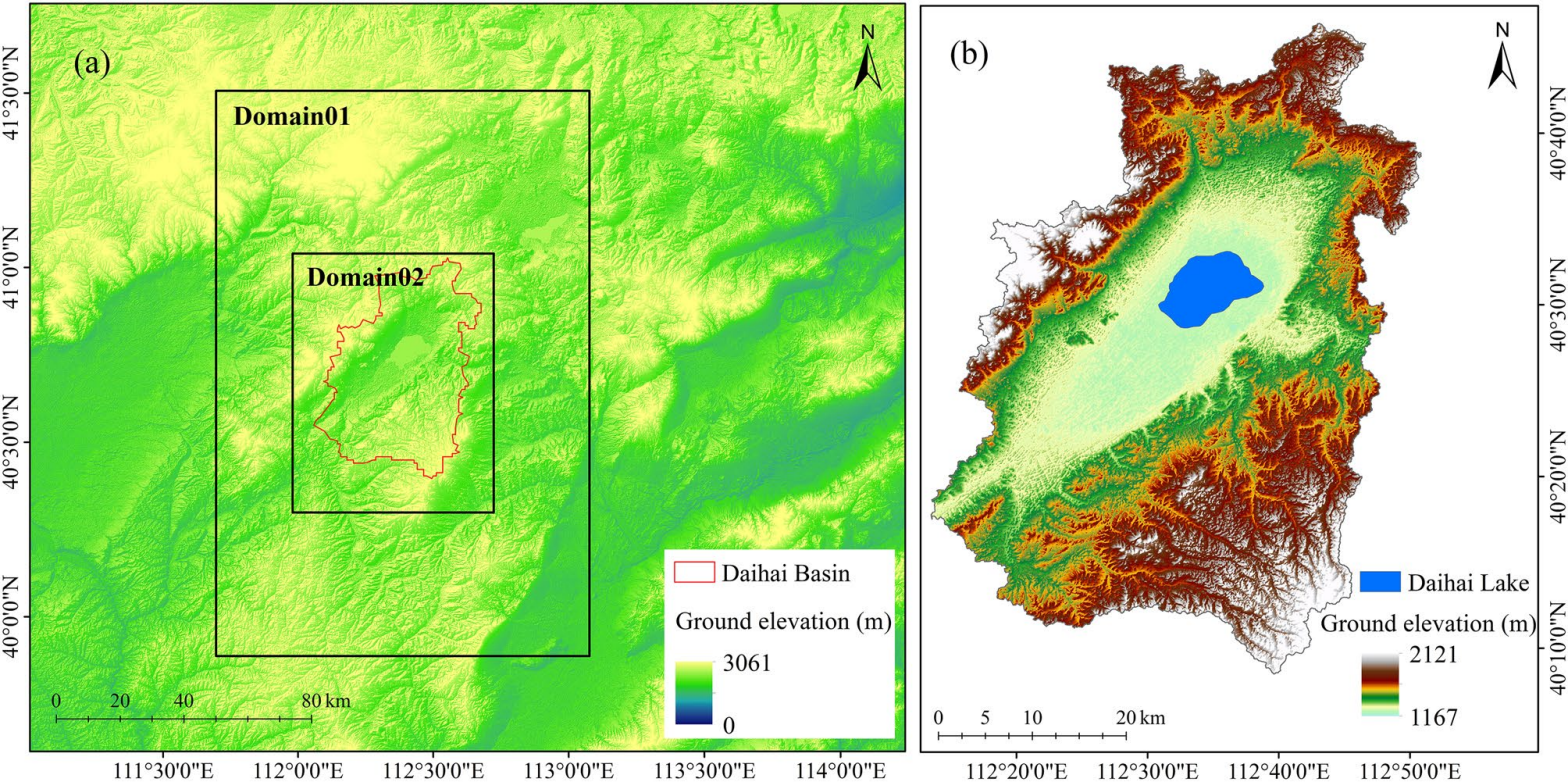
(**a**) Coverage and geographic location of spatially nested domains (**b**) Topographic of the Daihai Basin.

In this paper, WRF4.1.5 and WRF-Hydro5.1.1 online-coupling are used, and the basic settings are shown in Table 1. The coverage and geographical location of spatial nested domains are shown in Fig. 2 (produced by ArcGIS10.6, https://desktop.arcgis.com/de/arcmap/10.6/, the boundary of the control area is generated by the WRF preprocessing system, and the geographic elevation data is from NASA ASTERDEM.). According to the hydrological model and the size of the study area, the high-resolution precipitation product based on the downscaling of WRF model is more suitable for WRF-Hydro input. The most widely used physical parameter scheme in North China is adopted for physical parameters^23,24^, as shown in Table 2. To study the hydrological interaction lake and atmosphere in arid area, we turn on the lake option in the WRF-Hydro.

**Table 1:** Basic parameter settings of WRF and WRF-Hydro.

Model	Parameter	Setting
WRF	Domain horizontal resolution	D01: 3 km D02: 1 km
	Domain center	40°30′34.3′′N 112°38′45.5′′W
	Number of horizontal grids in the domain	D01: 40 × 60 D02: 64 × 82
	Scale	3:1
	Vertical layers	38
	Top pressure layer	50 hPa
	Integration time step	30 s
	Output time step	720 h
	Simulation time	1980/01/01 6:00–2021/01/01 6:00
	Land use data	MOD12Q1
	Leaf area index	MOD15A2
WRF-Hydro	Hydro output interval	720 h
	Surface model	Noah
	Re-interpolation factor	10:1
	Depth of soil column	2 m
	Roughness of soil layer	10 cm, 30 cm, 60 cm, 100 cm
	Built in resolution	100 m
	Model time step	10 s

**Table 2:** Physical parameters settings of WRF and WRF-Hydro.

Model	Parameter	Significance	Scheme selection
WRF	mp_physics	Microphysics scheme,	2, Purdue Lin scheme
	ra_lw_physics	Longwave radiation	1, RRTM scheme
	ra_sw_physics	Shortwave radiation	1, Dudhia scheme
	sf_surface_physics	Land surface scheme	2, Noah
	sf_lake_physics	Lake Physics	1, CLM 4.5 lake model
	bl_pbl_physics	Planetary boundary layer	1, Yonsei University scheme
	cu_physics	Cumulus parameterization	1, Kain-Fritsch scheme
WRF-Hydro	SUBRTSWCRT	Subsurface routing	1, Yes
	OVRTSWCRT	Overland flow routing	1, Yes
	CHANRTSWCRT	Channel routing	1, Yes
	channel_option	Channel routing option	3, Diff. Wave-gridded
	REFKDT	Runoff infiltration parameter	3
	RETDEPRTFAC	Surface retention depth scaling parameter	0.8
	OVROUGHRTFAC	Overland flow roughness scaling parameter	0.8
	MannN	Channel Manning roughness parameter	1.0
	GWBASESWCRT	Baseflow bucket model	0, No

The simulation time is 41 years from 6:00 on January 1, 1980 to 6:00 on January 1, 2021. The output step is adjusted to 720 h (30 days), that is, output on January 1, reducing the amount of calculation data. In addition, land use data are changed once a year and leaf area index is changed once a month to continuously adjust the impact of human activities on climate hydrological coupling in Daihai Basin.

#### Statistical analysis

1. Correlation matrix analysis

Correlation matrix, also known as correlation coefficient matrix, is composed of the correlation coefficients among the columns of the matrix^25^. When analyzing the driving factors of hydrological situation in Daihai Basin, the most influential factors can be selected from climate and human factors.

(2) Principal component analysis

Principal component analysis is to recombine highly correlated independent variables into a new set of independent principal components to replace the original variables, and all principal components are independent of each other^26,27^.

Generally, the first three principal components can reach more than 80% of all information. Principal component analysis can remove the redundant information between indicators and screen effective indicators. In this study, the principal component analysis method is used to screen out several important indicators which have an impact on the hydrological information of Daihai Basin. In addition, the contribution of human and nature to the hydrological information of Daihai Basin is distinguished.

### Data

#### Climate driven data

In this study, the initial and lateral boundary conditions of the parent region are defined by the reanalysis product (CFSR) of the climate prediction system of the National Center for environmental prediction (NCEP), with a spatial resolution of 0.5°. The time resolution is 6 h (00:00/06:00/12:00/18:00 UTC)^28^.

The high-resolution ground elevation data ASTERDEM used in WRF-Hydro GIS preprocessing system is from NASA with a spatial resolution of 30 m.

The land use data in WPS geo spatial static database was replaced by MODIS MOD12Q1 product year by year, and the leaf area index data was replaced by MOD15A2 data month by month (Table 1).

All the time in this study was calculated and described in universal time.

#### Validation data

The precipitation verification data uses the year-by-year observation data from Liangcheng Station, and the precipitation spatial distribution verification data comes from the interpolation products calculated and processed by the Resource and Environmental Science and Data Center of the Chinese Academy of Sciences (China's annual precipitation spatial interpolation data set since 1980, resolution 1 km^29^, http://www.resdc.cn/data.aspx?DATAID=229). There is no hydrological station for perennial surveys in the Daihai Basin, and there is a lack of runoff verification data in the Daihai Basin. Remote sensing estimation method is used to calculate the annual inflow of Daihai Lake to verify the output variable of WRF-Hydro model.

The calculation unit of runoff into the lake in WRF-Hydro is m^3^/s, while the calculation unit of remote sensing estimation is m^3^. Simple conversion of units is required, as shown in the Eq. 2.

1 $$V = vT$$

where, V is the amount of water into the lake, in m^3^, v is the runoff velocity, in m^3^/s, T is the runoff time, in s.

The basic principles of remote sensing estimation are as follows: according to the view of water balance, the rise or fall of lake water level is caused by the imbalance of lake water budget^30^. Daihai Lake is an inland closed lake. The water revenue of the lake includes Lake precipitation, surface and underground runoff, including Lake evaporation and lake water intake^31^. Daihai has serious eutrophication, water quality exceeds the standard, and there is no large amount of lake water intake, so the lake water intake item can be ignored^30^. Therefore, the water balance of Daihai can be expressed as Eq. 3^30,31^:

2 $$\Delta V = P - E + R = (H_{2} - H_{1} )S$$

where, ∆V is the annual change of lake water quantity, P is the annual precipitation of lake water surface, E is the annual evaporation of lake water surface, R is the surface and underground runoff into the lake, H_1_ and H_2_ are the lake water level at the beginning and end of the year respectively, and S is the lake area.

The annual precipitation and evaporation of the lake surface are measured by Liangcheng station which is not far from Daihai. Generally, the precipitation and evaporation are in depth unit mm, and the volume is in the formula, so it needs to be converted according to the lake area. Lake area is the average value of Lake area at the beginning and end of the year, which is obtained by visual interpretation and digitization of remote sensing images at the beginning and end of the year. According to the lake boundary at the beginning and the end of the year obtained from remote sensing interpretation and DEM of digital elevation model, the lake change water quantity is obtained by superposition analysis and volume analysis in ArcGIS. Therefore, according to the above calculation and collation, the calculation formula of the discharge into the lake is shown in the^31^.

3 $$R{ = }\Delta V{ - }P{ + }E$$

Landsat TM, ETM and OLI are the main remote sensing images used in the calculation, with a resolution of 30 m, which are from NASA.

### Output correction settings

In the process of climate hydrological coupling of long time series in Daihai Basin, the differences between the coupling results and the measured results may be caused by the quality of climate driven data, the lack of model parameters and human impact. Therefore, it is necessary to partially correct the long-time coupling results to minimize the human activities impact. The revised output is the runoff product into the lake. It is assumed that the difference between the runoff estimated by remote sensing and the simulated runoff comes from human impact, including agricultural irrigation water and industrial water. Therefore, the difference between the multi-year remote sensing estimation and the runoff into the lake can be determined by establishing multiple linear correlation with agricultural irrigation water consumption and industrial water consumption. Then the modified runoff difference is calculated from the agricultural irrigation water and industrial water in the current year, and the modified simulated runoff is calculated from the difference runoff and the original runoff.

The agricultural irrigation water and industrial water used in the correction are from the local statistical yearbook.

## Results

### Coupling accuracy analysis

#### Precipitation simulation accuracy

**Figure 3:**
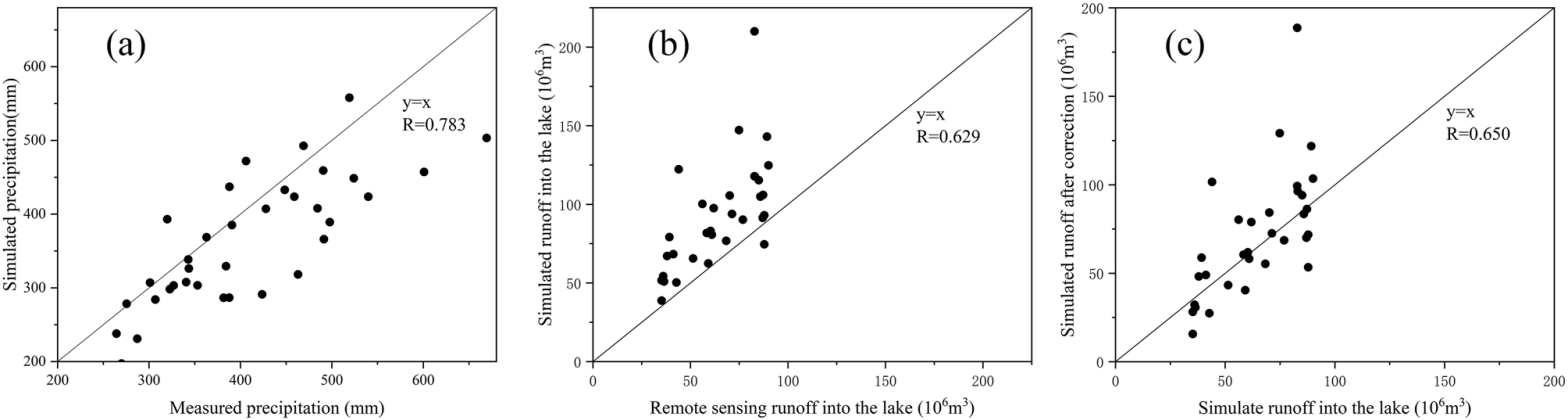
(**a**) Comparison between WRF-HYDRO simulation and measured annual precipitation in Daihai; (**b**) Comparison of runoff simulation and remote sensing estimation in Daihai Lake; (**c**) Modified runoff simulation and remote sensing estimation in Daihai Lake.

The comparison between annual precipitation simulated by WRF-Hydro and measured precipitation is shown in the following Fig. 3a. From the Fig. 3a, we can get that the correlation between simulated precipitation and measured precipitation is 0.783, which is relatively high and the simulation is good. In addition, the simulated precipitation is less than the measured precipitation value in time. We guess that this error is caused by the precision and quality of precipitation products. WRF-Hydro can easily underestimate the duration of heavy rain when simulating precipitation, so the simulated precipitation is slightly smaller than the measured precipitation in long-term sequence, but the overall accuracy is good.

**Figure 4:**
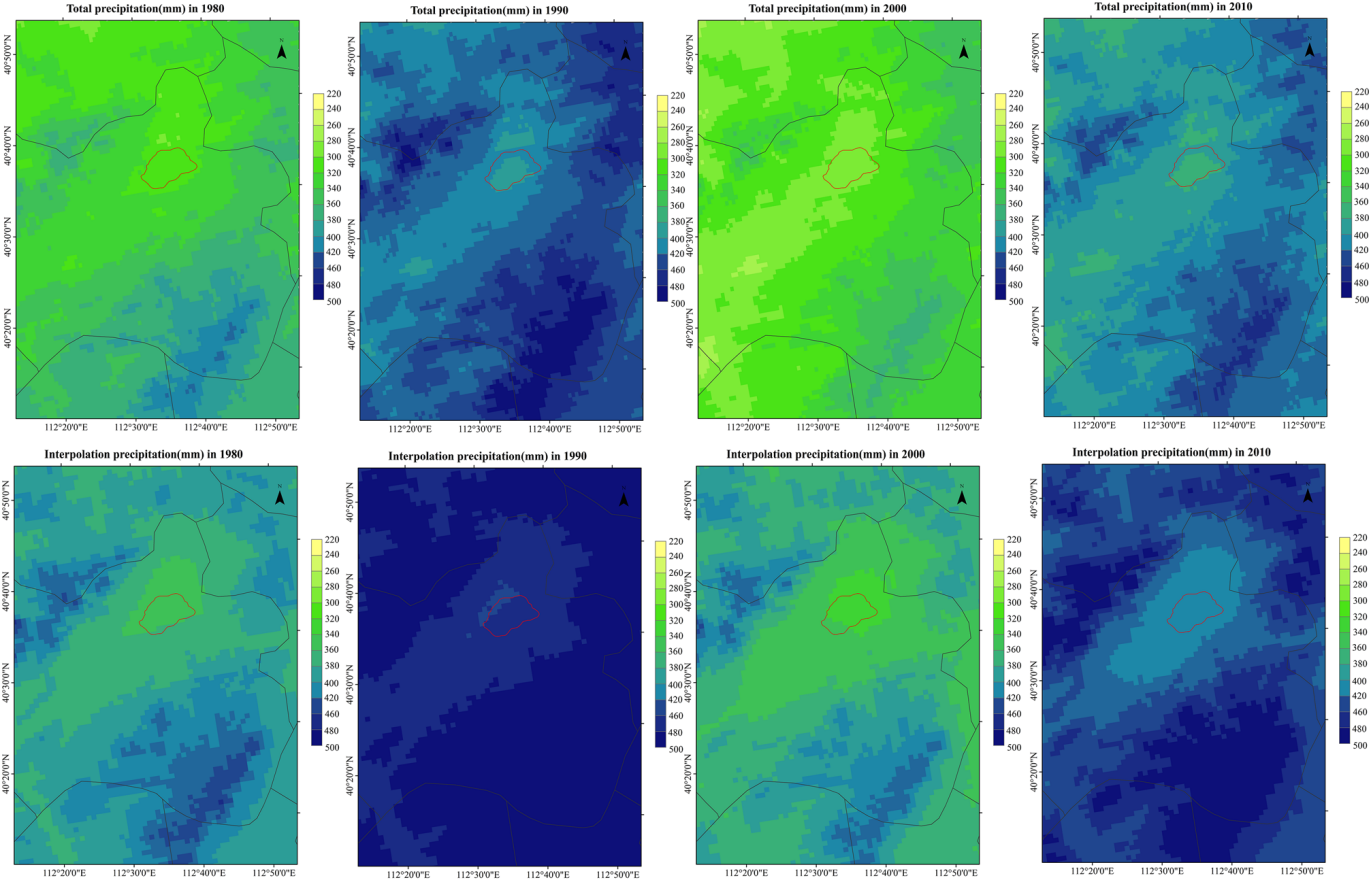
Spatial comparison of WRF-HYDRO simulation and interpolation of annual precipitation in Daihai.

The comparison between the simulated spatial distribution of annual precipitation and the verified products in the study area is shown in the Fig. 4. Generally speaking, the precipitation of interpolation products is slightly higher than the simulation value, which is consistent with the above analysis. In addition, the spatial distribution law of the two is consistent with each other, and the spatial variation law is basically the same. However, the transition of simulation results in areas with severe precipitation changes is relatively gentle, while the transition of interpolation products is more severe. The coverage of the maximum value in the simulation results is smaller than that of interpolation products. The guess is caused by the error of setting the precipitation boundary line. The boundary of interpolation products is China as a whole, and the boundary of simulation results is only Daihai Basin, which fundamentally determines that the precipitation simulation results will be slightly smaller than the interpolation products. Because the climate and hydrology mutual chamber is defined in the model setting from the surrounding grid points, the smaller the area causes some areas with mutual chamber cannot enter the boundary line, resulting in the precipitation simulation results less than the interpolation products. But in terms of the overall spatial differentiation law, the distribution of simulation results in interpolation products is not very different, which has good practical value.

#### Simulation accuracy of runoff into Lake

The comparison between the WRF-Hydro simulation results and remote sensing estimation results of the runoff from Daihai Lake for many years is shown in the Fig. 3b. It can be seen from the figure that the correlation between simulation results and remote sensing estimation results is 0.629, which is better. But it is obvious that the simulation results are higher than those of remote sensing. The reason may be that the model does not set up the parameters of man-made water from the river entering the lake, including agricultural irrigation water and industrial water intake. So the simulation results are overestimated to the runoff into the lake. Therefore, the simulated runoff into the lake is modified in this study to reduce the water consumption ignored by the model.

The comparison between the revised simulated runoff and remote sensing estimation is shown in the Fig. 3c. As can be seen from the figure, the correlation is increased to 0.650. Although not much improvement, the simulation results and remote sensing results are distributed evenly around the boundary.

### Analysis of coupling results

#### Precipitation analysis

**Figure 5:**
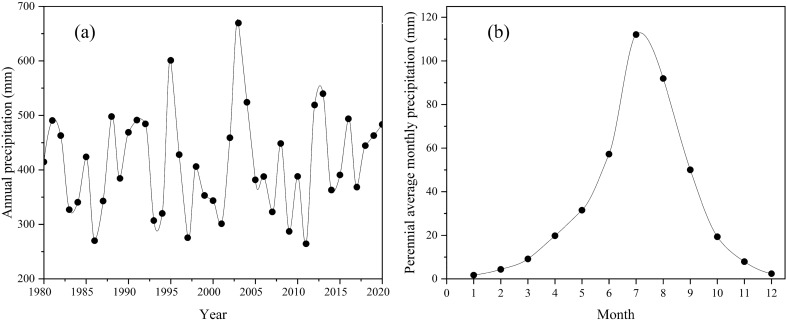
(**a**) Distribution curve of annual precipitation in Daihai Basin; (**b**) Distribution curve of annual mean monthly precipitation in Daihai Basin.

The precipitation in Daihai Basin is relatively abundant. Except for some extreme drought years and humid years, the average annual precipitation is 300–600 mm (see Fig. 5a), and the average annual precipitation is about 400 mm. It can be seen from the figure that the minimum annual precipitation is less than 250 mm; The maximum annual diameter is higher than 750 mm. The difference between extreme dry year and extreme wet year is three times.

The monthly average of precipitation in the Daihai Basin for many years is shown in the Fig. 5b. It can be seen from the figure that the precipitation in the Daihai Basin is unevenly distributed throughout the year, with the least in January at 1.73 mm and the most in July at 112.10 mm. The precipitation in July–August accounts for more than 50% of the total annual precipitation. In addition, it can be seen from the figure that the precipitation in the Daihai Basin is mainly concentrated in June to September, which is also the flood season in the Daihai Basin, accounting for more than 70% of the total annual precipitation.

Combined with Table 3, overall, the average precipitation from 1980 to 1994 is 401.75 mm, with little fluctuation; During the period from 1995 to 2011, except for extreme precipitation in some years (more than 600 mm in both 1995 and 2003), the precipitation decrease, with an average value of 371.39 mm. There are several dry years and wet years, and the fluctuation range was sharp; From 2012 to 2020, the fluctuation range is small, and the average value rises to 451.75 mm.

**Table 3:** Average precipitation (mm) in different periods in Dahai Basin

Periods	1980–1994	1995–2011	1995–2011(Excluding 1995 and 2003)	2012–2020
Average precipitation	401.75	402.42	371.39	451.75

**Figure 6:**
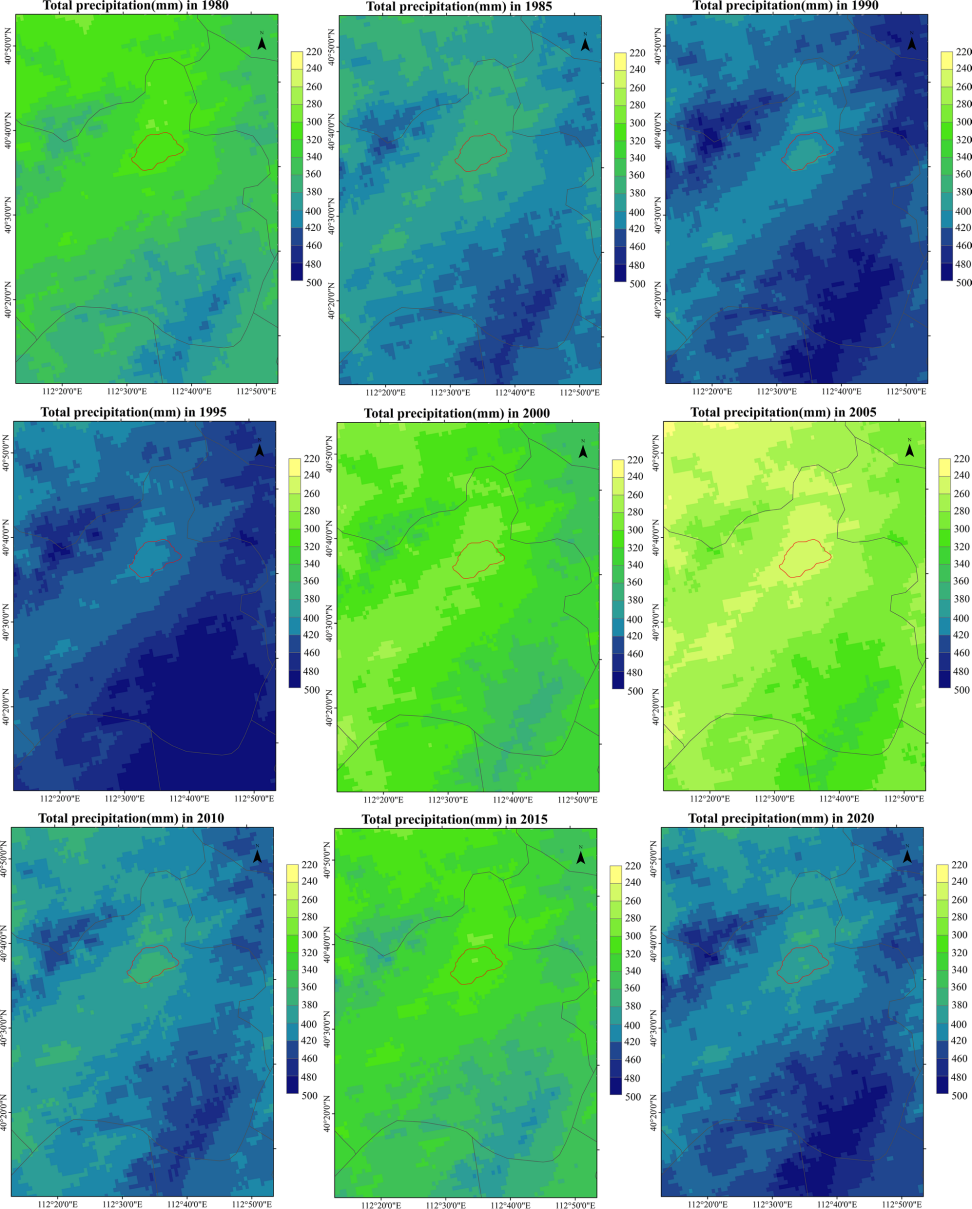
Spatial distribution of annual precipitation in Daihai Basin.

The spatial distribution of annual precipitation in Daihai Basin is shown in the Fig. 6. It is obvious from the figure that the precipitation in 1990, 1995 and 2020 is abundant compared with other years. In addition, it is found that although the annual precipitation in Daihai Basin varies in size, its spatial distribution is basically the same.

The spatial pattern of annual precipitation in Daihai Basin is as follows: the southeast of Liangcheng County and the north of Zuoyun County, the northwest of Liangcheng County and the northwest of Fengzhen county are the three precipitation centers, which gradually decrease outward. And the central effect of Fengzhen county is not obvious in some years. In addition, it is found that the area around Daihai Lake has the least precipitation in the whole Daihai Basin. This may be related to the terrain surrounding the Daihai Basin.

In the whole study area, the annual precipitation in the north of Zuoyun County is larger than that in other regions. In some years, the annual precipitation reaches 800 mm, and the extension area is wide. In some years, it extends to the southeast of Liangcheng County. Therefore, it is speculated that mountain torrents, debris flows, rainstorms, snowstorms and other natural disasters are prone to occur here.

In addition, combined with the topographic map, it is found that the southeast and northwest of Liangcheng County are the highest elevation in the study area, which coincides with the extreme precipitation. At the same time, it is found that the spatial consistency of precipitation distribution in the whole study area is higher than that of terrain distribution in the study area. Therefore, it is speculated that the precipitation in the study area is seriously affected by the terrain, in other words, the precipitation in the study area is mostly terrain rain or mountain convective rain.

#### Runoff analysis

**Figure 7:**
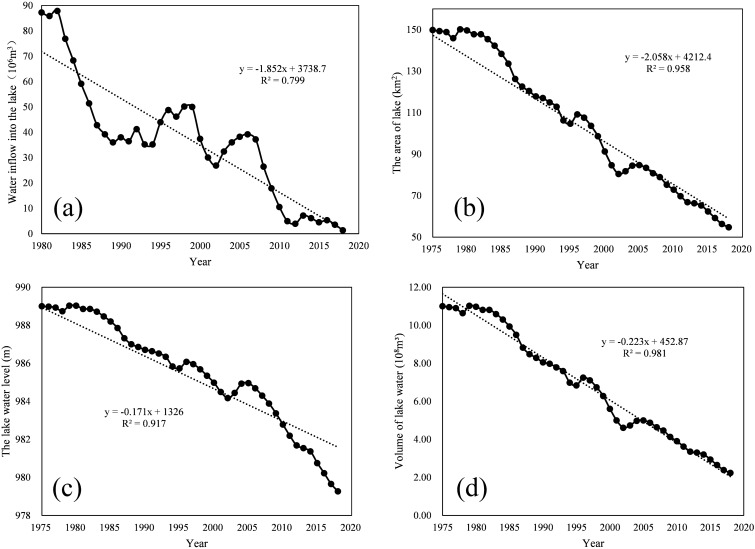
(**a**) Change of runoff in Daihai Lake over the years; (**b**) Changes of lake area in Daihai over the years; (**c**) Changes of lake water level in Daihai over the years; (**d**) Changes of volume water in Daihai Lake over the years.

The Runoff Curve of Daihai Lake is shown in the Fig. 7a. It can be seen from the figure that the flow into the lake shows a downward trend from 1980 to 2020. Although it rebounded in 1996–1999 and 2005–2007, after 2010, the runoff into the lake decreased sharply below 8 × 10^6^m^3^. From 1980 to 1990, the runoff into the lake decreased linearly with a larger slope and a faster speed; However, from 1990 to 2000, the runoff into the lake appeared the first vibration wave peak, and from 2000 to 2007, the second vibration wave peak. From 2008 to 2012, the decline rate was sharp, and the runoff into the lake had been reduced to 3.95 × 10^6^m^3^ in 2012; Since 2013, the runoff into the lake tends to be flat, but it has not exceeded 10 × 10^6^m^3^.

The change curve of Daihai Lake area is shown in the Fig. 7b. It can be seen from the figure that the area of Daihai Lake is declining in a straight line. In a short period of 40 years, the lake area has shrunk nearly 100 km^2^. In addition, we found that the shrinkage rate of Daihai Lake area slowed down from 1980 to 1985, but the lake area shrank sharply from 1995 to 2000. After 2005, the atrophy curve almost coincided with the fitting curve, and the overall fitting R^2^ was as high as 0.958.

The water level variation curve of Daihai Lake is shown in the Fig. 7c. As can be seen from the figure, the variation trend of water level in Daihai Lake is very similar to that of lake area. However, the slope of lake water level change is less than the change rate of lake area. In the 40 years since 1975, the water level in Daihai has dropped by nearly 10 m. In addition, the water level rose slightly in 1995–1996 and 2003–2006. And after 2006, Daihai water level decline rate also accelerated. Since 2006, the water level of Daihai has dropped nearly 6 m, with a rate of 0.45 m/year.

The trend of the volume water volume of the Daihai Lake is shown in the Fig. 7d. It can be clearly seen from the figure that the decline curve of the Daihai Lake water volume is close to a straight line, especially from 2005 to the present, the fitting degree is as high as 0.981. There should be some geometrical relationship among the lake area, water level and water volume, and this relationship should be related to the digital elevation model of the lake bottom. In addition, the changes of lake bottom topography are not linear, so there are still subtle differences between the three changes.

**Figure 8:**
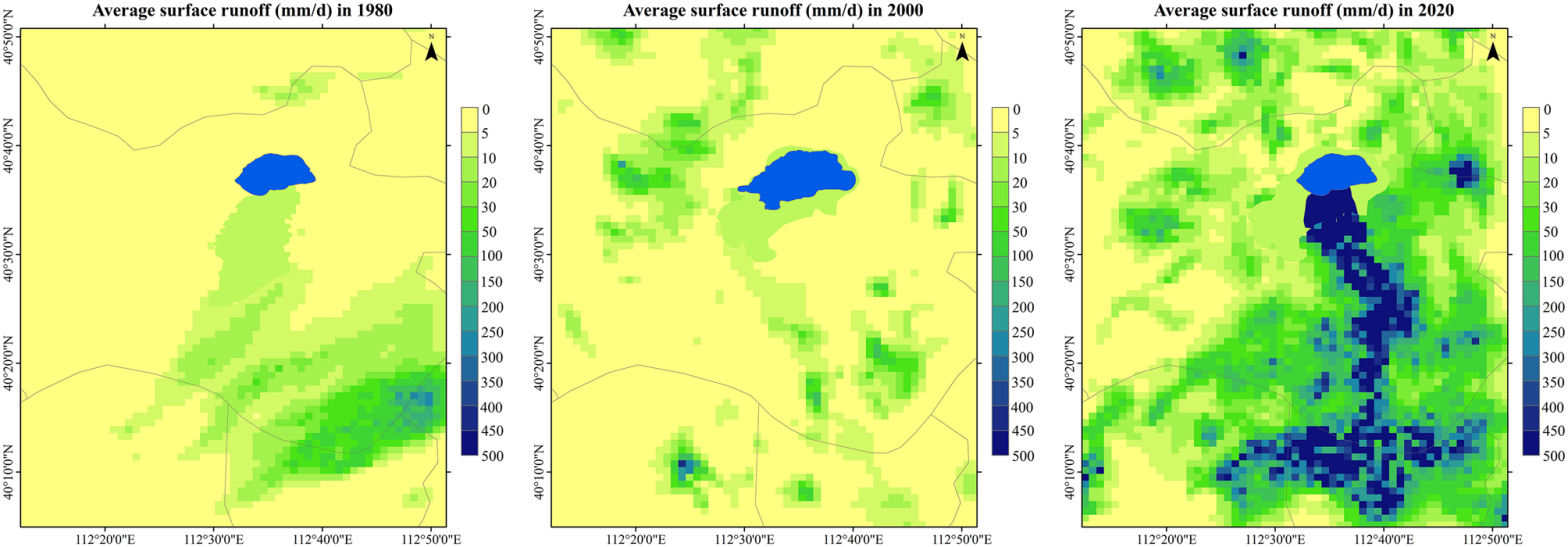
Spatial distribution of surface runoff in Daihai Basin.

The annual surface runoff of Daihai Basin is shown in the Fig. 8. It can be seen from the figure that the Gongba River, the Wuhao River, the Buliang River and the Tiancheng River in the south of Daihai Lake supply the Daihai Lake for a long time, and the Bantanzi River in the West also flows into the Dai sea in some years. Combined with the spatial distribution of annual precipitation, it can be concluded that surface runoff is seriously affected by precipitation. The annual distribution is uneven. The surface runoff from the southeast of Liangcheng County generally flows into Daihai Lake to the north, but in some drought years, it will be stopped and cannot flow into Daihai Lake. Bantanzi River in the west of Daihai Lake also supplies Daihai Lake in the year of more precipitation.

**Figure 9:**
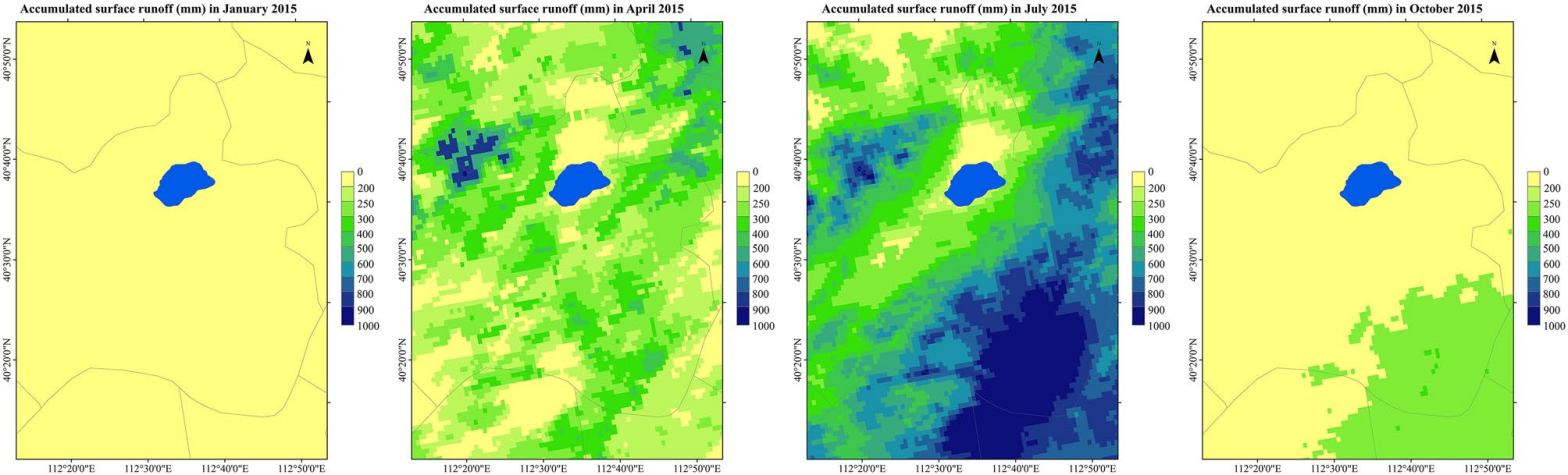
Spatial distribution of surface runoff in different seasons in Daihai Basin.

Taking the surface runoff of Daihai Basin in January, April, July and October 2015 as an example, the distribution of surface runoff in different seasons of the year is analyzed, as shown in the Fig. 9. It can be seen from the figure that the rivers in Daihai Basin are seasonal rivers, which are prone to be cut off in autumn and winter. In winter (December–February), there will be different degrees of snowfall events in Daihai Basin, but due to the river freezing period and small snowfall, there will be no runoff. In spring (March to May), the precipitation in Daihai Basin began to increase, and the surface runoff also began to increase, mainly from the southeast and northwest of Liangcheng County. Gongba River, Wuhao River, buliang River, Tiancheng River and Bantanzi River in the south of Daihai Lake will supply Daihai Lake, but these rivers have small flow in spring, which is easy to break. Summer (June–August) is the main period of precipitation in Daihai Basin, and the surface runoff will also surge. In July 2015, the runoff in some areas reached 2000 mm, which was prone to flood disaster. The rivers in the west and south of Daihai Lake will supply it, but the runoff into Daihai Lake is not high, and most of the runoff is concentrated in the upper and middle reaches. In autumn (from September to November), the precipitation in Daihai Basin decreases. Before the freezing period, the precipitation may form runoff, but it is difficult to flow into Daihai Lake due to the small flow.

### Statistical analysis of other factors

#### Climatic factors

1. Evaporation capacity

**Figure 10:**
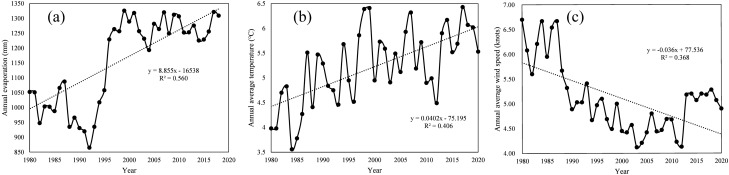
Perennial (**a**) evaporation (**b**) annual average temperature (**c**) annual average wind speed change in Daihai Basin.

The variation curve of annual evaporation in Daihai is shown in the Fig. 10a. It can be seen from the figure that although the evaporation in Daihai Basin fluctuates, it shows an upward trend, with an upward slope of 8.855 and R^2^ of 0.560. From 1980 to 1986, the annual evaporation fluctuated around 1000 mm; From 1987 to 1992, the annual evaporation of Daihai Basin decreased sharply, but from 1993 to 2000, the annual evaporation increased sharply with a very high rate of increase; But after 2000, the annual evaporation fluctuated and remained at 1250 mm.

(2) Average temperature

The variation curve of annual average temperature in Daihai is shown in the Fig. 10b. It can be seen from the figure that the annual average temperature in Daihai Basin presents an obvious fluctuating upward trend, and the fitting upward slope is 0.040, R^2^ is 0.406. In addition, it can be observed that in a 10-year cycle, there will be two small fluctuations and one large fluctuation, and the fluctuation will rise.

(3) Wind speed

The curve of annual average wind speed in Daihai is shown in the Fig. 10c. It can be seen from the figure that the annual average wind speed of Daihai Basin presents a fluctuating downward trend, and the fitting downward slope is 0.036, R^2^ is 0.368. In addition, it can be observed that the annual average wind speed fluctuated with a mean line of 6.2 from 1980 to 1987; In 1988 and 1990, it dropped sharply with a large slope; From 1990 to 2003, the fluctuation decreased. From 2003 to 2011, the fluctuation was stable at 4.5, and rose sharply in 2012. So far, the fluctuation has been stable at 5.2.

#### Human factors

1. Cultivated land area

**Figure 11:**
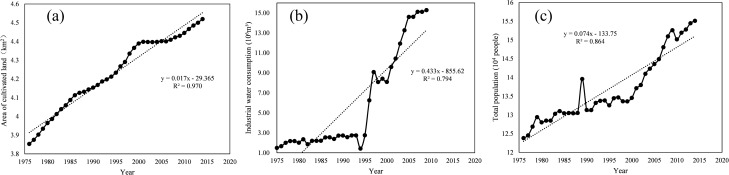
Perennial (**a**) cultivated land area (**b**) industrial water consumption (**c**) total population change curve in Daihai Basin.

The change curve of cultivated land area in Daihai Basin is shown in the figure. It can be seen from the Fig. 11a that the annual average wind speed in Daihai Basin presents an upward trend, with the fitting rising rate of 0.017 and R^2^ of 0.970, almost in a straight line. In addition, it can be observed that from 1996 to 2005, the rising rate appeared a trough, that is, the rising rate first increased rapidly and then decreased. From 2000 to 2005, the rising rate was very slow and approached zero; But since 2006, it has returned to a straight-line rise.

(2) Industrial water consumption

The change curve of industrial water consumption in Daihai Basin is shown in the Fig. 11b. It can be seen from the figure that the industrial water consumption of Daihai Basin presents an upward trend, and the fitting rising rate is 0.433, R^2^ is 0.794. In addition, it can be observed that from 1975 to 1993, the industrial water consumption of Daihai Basin was below 3 × 10^6^m^3^; From 1994 to 2005, except for the decrease in 1998–2000, it has been on the rise, and the rising speed is fast, which has increased five times in ten years; Since 2005, the industrial water consumption in Daihai Basin has been stable at about 15 × 10^6^m^3^.

(3) Total population

The change curve of total population in Daihai Basin is shown in the Fig. 11c. It can be seen from the figure that the total population of Daihai Basin presents an upward trend, and the fitting rising rate is 0.074, R^2^ is 0.864. In addition, it can be observed that the total population of Daihai Basin increased slowly from 1975 to 1985; From 1986 to 1990, the total population remained flat; It fluctuated from 1990 to 2000; Since 2000, the total population has risen sharply.

### Analysis of driving factors of hydrological information

In this study, the average temperature, annual precipitation, annual evaporation, average wind speed in natural factors and cultivated land area, agricultural water consumption, industrial water consumption and population in human factors are considered as the influencing factors of runoff change in Daihai Lake. Therefore, the flow into the lake and the above elements constitute a variable sequence, and the correlation matrix is calculated. See the Table 4 for details.

**Table 4:** Correlation matrix between lake inflow and influencing factors.

Correlation	Lake inflow	AWC	IWC	Population	CLA	AP	AE	AWS	AT
lake inflow	1								
AWC	0.276	1							
IWC	− 0.544**	− 0.399*	1						
Population	− 0.660**	− 0.271	0.893**	1					
CLA	− 0.777**	− 0.384*	0.887**	0.796**	1				
AP	0.016	− 0.352	− 0.223	− 0.201	− 0.147	1			
AE	− 0.371*	− 0.230	0.848**	0.633**	− 0.813**	− 0.345	1		
AWS	0.690**	0.415*	− 0.707**	− 0.647**	− 0.862**	0.133	− 0.567**	1	
AT	− 0.530**	− 0.055	0.579*	0.518**	0.694**	0.274	0.605**	− 0.654**	1

AWC is agricultural water consumption; IWC is industrial water consumption; CLA is cultivated land area; AP is annual precipitation; AE is annual evaporation; AWS is average wind speed; AT is average temperature; ** is significantly correlated at 0.01 level, *is significantly correlated at 0.05 level.

It can be seen from the Table 4 that the cultivated land area has the highest correlation with the runoff into the lake, with a correlation of − 0.777, which is highly significant, followed by the wind speed, with a correlation of 0.690, which is highly significant; In addition, the total population, industrial water consumption, evaporation and average temperature were significantly correlated. Therefore, the discharge of Daihai Lake is influenced by both nature and human. It can be seen from the table that industrial water consumption, total population, cultivated land area, evaporation and annual average temperature have a negative impact on the flow into the lake, while wind speed has a positive impact.

At the same time, the correlation between different factors can be obtained from the Table. For example, the correlation between industrial water consumption and population, cultivated land area and evaporation is as high as 0.8, which is highly significant; The correlation between population and cultivated land, cultivated land and wind speed and evaporation is also about 0.8, which is highly significant; In addition, the correlations between industrial water consumption and annual average temperature, population and annual average temperature, wind speed, evaporation, cultivated land, cultivated land and annual average temperature, evaporation and wind speed, wind speed and annual average temperature are all over 0.5.

It can be clearly observed from the table that except for agricultural water consumption, precipitation and evaporation, the annual average temperature is significantly correlated with other factors, and the correlation is more than 0.5. The correlation between annual precipitation and other factors is small and not significant. Therefore, it can be determined that there is data redundancy between different elements. In order to eliminate the data redundancy and get the determinants of the discharge into the lake, the correlation analysis of the variable sequence is carried out, as shown in the table.

It can be seen from the Table 5 that the cumulative variance of the first three principal components has reached 87.016%, and the eigenvalues of the first two principal components are greater than 1, which has met the standard. The variance contribution rate of the first principal component was 59.641%, and the order of load rate was cultivated land (0.967), industrial water (0.950), population (0.859), evaporation (0.856), wind speed (0.841), and the load rate was greater than 0.8; In the first principal component, the influence of human factors is greater than that of natural factors. In the second principal component, the variance contribution rate is 18.821%, in which the annual precipitation (− 0.875) and agricultural water consumption (0.736) have higher load rate, and the influence of natural factors is greater than that of human factors.

**Table 5:** Component matrix of principal component analysis of different influencing factors

Factor	PC1	PC2	PC3
AWC	− 0.397	0.736	0.377
IWC	0.950	0.011	− 0.223
Population	0.859	0.037	− 0.163
CLA	0.967	− 0.049	0.054
AP	− 0.205	− 0.875	0.291
AE	0.856	0.213	− 0.162
AWS	− 0.841	0.296	− 0.336
AT	0.746	0.248	0.490
The eigenvalue	4.771	1.506	0.684
Variance contribution rate	59.641%	18.821%	8.554%
Cumulative variance contribution rate	59.641%	78.463%	87.016%

AWC is agricultural water consumption; IWC is industrial water consumption; CLA is cultivated land area; AP is annual precipitation; AE is annual evaporation; AWS is average wind speed; AT is average temperature; PC1 is the first principal component; PC2 is the second principal component; PC3 is the third principal component.

### Future forecast

According to the analysis in Sect. 3.4, we find that human factors have a huge impact on the lake inflow. In lake water balance, precipitation and evaporation are determined by climate. Now, the Inner Mongolian government has taken a series of measures to protect the Daihai Lake. Therefore, when we predict the future lake water volume, we consider two situations: (1) the future lake water volume in the natural state without any interference (protection or destruction) measures; (2) keeping the existing water volume unchanged future lake water volume in the case.

#### Situation I

For the Situation I, we use two forecasting methods. Method I is to directly predict the future lake water volume by using the variation law of lake volume water volume with time. Method II is to use the lake water balance equation to estimate the change in lake water volume, and then estimate the future lake water volume. The results obtained by these two calculation methods are shown in the Table 6.

**Table 6:** Future prediction of Daihai Lake in situation I.

Year	Method I			Method II	
	Water volume (10^9^ m^3^)	Lake area (km^2^)	Lake depth (m)	Lake inflow (10^6^m^3^)	Water volume (10^9^ m^3^)
2021	1.862	52.778	12.655	8.741	3.458
2022	1.658	50.720	12.485	6.099	3.162
2023	1.453	48.661	12.314	3.456	2.867
2024	1.249	46.603	12.143	0.814	2.571
2025	1.045	44.545	11.973	− 1.829	2.275
2026	0.840	42.487	11.802	–	1.980
2027	0.636	40.429	11.631	–	1.684
2028	0.431	38.370	11.460	–	1.388
2029	0.227	36.312	11.290	–	1.092
2030	0.023	34.254	11.119	–	0.797
2031	− 0.182	32.196	10.948	–	0.501
2032	–	30.138	10.778	–	0.205
2033	–	28.079	10.607	–	− 0.090
2046	–	1.323	8.388	–	–
2047	–	− 0.735	8.217	–	–
2095	–	–	0.024	–	–
2096	–	–	− 0.147	–	–

When estimating the dry years of the Daihai Lake, the results obtained by using the time-varying laws of lake area, water volume and lake depth are inconsistent. Among them, the dry year of the Daihai Lake obtained by using the water volume is 2031, the lake area is 2047, and the water depth is 2096. The three are vastly different. The reason is the uncertainty of our modeling data. As Daihai Lake is a lake in an arid area, data is extremely scarce, and there is almost no continuous measurement of water level, depth, and water volume. The lake area is interpreted from remote sensing images and is an annual average, which results in neglect of inter-annual hydrological changes. Similarly, the water depth is also obtained by remote sensing. The resolution of the remote sensing image is 30 m. We use the interpolation method to control the accuracy to about 5 m. However, in the later stage of the prediction, when the lake depth is lower than 10 m, the results begin to become inaccurate. The modeling data of lake water volume were obtained from WRF-Hydro simulations, so the uncertainty of the data led to the inconsistency of the results. We choose the most recent year as the final result of method I, that is, the forecast result of water volume.

From the Table 6, we can observe that the calculation results of the two methods are quite different. The reason is that in method I, we assume that the volume of water in the lake changes linearly, and there is only one variable; in method II, the number of variables increases and the uncertainty increases. However, the years when the Daihai Lake is predicted to dry up are basically the same. Method I predicts that the Daihai Lake will be depleted in 2031, and method II is 2033, which is not much different.

#### Situation II

For the situation II, we control the agricultural water consumption and industrial water consumption to remain unchanged, estimate the change of volume water at this time, and then estimate the future lake water volume. Among them, the change in water consumption is only evaporation, and the change in water replenishment is precipitation and runoff. The future lake inflow and lake water volume calculated by using the water balance equation are shown in the Table 7:

**Table 7:** Future prediction of Daihai Lake in situation II.

Year	Lake inflow (10^6^m^3^)	Water volume (10^9^ m^3^)
2021	11.094	3.721
2025	9.938	3.591
2030	8.492	3.430
2040	5.601	3.107
2050	2.709	2.783
2060	− 0.182	2.460
2070	–	2.136
2080	–	1.812
2090	–	1.489
2100	–	1.165
2110	–	0.842
2120	–	0.518
2130	–	0.195
2140	–	− 0.129

From the Table 7, we can see that under human control, although the of lake inflow will continue to decline compared with no measures, the rate of decline will be significantly slower. And the lake inflow will drop to 0 in 2060. Similarly, the water volume in the Daihai Lake will decline. But the rate is significantly slower compared with situation I. And the water volume will drop to 0 in 2140, nearly 110 years later than 2032–3033 without any control. This shows that man-made protection of the Daihai Lake is extremely important.

## Discussion

Climate change and human activities are the decisive factors leading to the change of sea water volume and Daihai Lake area in^32^. Among them, human activities play a greater role. With the increase of population, the area of cultivated land is gradually expanding, and the industrial water demand and irrigation water are also increasing. The water intake and precipitation interception of the rivers in Daihai Basin will also increase, and Daihai Lake can not get effective supply, and its area is gradually decreasing^33^. Therefore, the correlation among population, cultivated land area, industrial water demand and inflow is negative. Although cultivated land irrigation has the highest correlation with the flow into the lake and has the greatest impact, with the increase of policies and measures to protect Daihai Lake and "returning farmland to Grassland", cultivated land area is controlled and agricultural water consumption is controlled^32^.

Climate is the second major factor affecting the discharge of Daihai Lake^34^. Daihai Lake is an inland closed lake, which is mainly supplied by precipitation and runoff. However, the correlation between Daihai Lake discharge and precipitation is small and not significant. The main reason is that runoff generated by precipitation is consumed and utilized by human beings^32^. In addition, the correlation between the discharge into Daihai Lake and the annual average temperature and evaporation is negative, mainly due to the increase of temperature and evaporation, and the discharge into Daihai Lake is evaporated into the air before entering Daihai Lake^30,35^.

The comparison of the predictions under the two different scenarios just shows that human protection measures are extremely important^36,37^. If human activities are not controlled and managed, the Daihai Sea will dry up in 2031–2033, a year that is still ten years away, which is very shocking. But in the second case, Daihai still has 120 years to live. In other words, if human activities take stronger protective measures on this basis, the lifespan of Daihai Lake may be extended.

Now it is necessary to take measures to protect the Daihai Basin. According to the analysis of the impact on the discharge of Daihai Lake, the cultivated land area and industrial water use have a very serious impact on the discharge of Daihai Lake. Therefore, the measures of returning farmland to forest and grassland are of great value. In addition, it is necessary to popularize water-saving irrigation technology in Daihai Basin. In order to protect the Daihai River Basin, a reservoir can also be built in the Daihai Basin to store precipitation to replenish the Daihai Lake regularly and increase the man-made water consumption, without taking water directly from the runoff into the lake.

Due to the influence of time and equipment, the research on WRF-Hydro long-term climate hydrological coupling is not in-depth. In the future, we can improve the climate hydrological coupling model of long time series from the following aspects

1. In arid and semi-arid areas, the parameter deviation of WRF-Hydro can be eliminated by modifying or canceling the bucket model of WRF-Hydro base flow to allow deep groundwater recharge in arid and semi-arid areas^38^. Reasonable application of groundwater model can obtain better runoff simulation results. Considering the limitation of the basic flow bucket model in WRF-Hydro, coupling WRF-Hydro with groundwater model may be helpful to the accuracy of runoff simulation in the future.
2. Through multi-source data assimilation and the integration of weather radar and numerical weather forecast model, the accuracy of precipitation forecast and the results of hydrological forecast can be improved to a certain extent. The further improvement and development of precipitation forcing accuracy and its application in hydrological forecast are the important trend of hydrometeorological forecast in the future^39^. At present, the application of hydrological forecast is mainly based on the unidirectional coupling of Meteorology and hydrology. As the core of precipitation and runoff conversion, WRF-Hydro strengthens the research on the physical parameter values, parameter calibration and uncertainty of precipitation forcing, in order to explore the uncertainty of atmospheric hydrological coupling model under regional atmospheric variability and produce more realistic hydrological response.
3. In arid and semi-arid areas, some lake basins are severely affected by human activities, such as Daihai Basin. In the process of long-term climate hydrological coupling, the simulated value is higher than the actual value because of the influence of human activities (agricultural water, industrial water, domestic water, etc.). Therefore, the human influence module should be added to the climate hydrological coupling model to reduce the error of human activities responding to the climate hydrological interaction.

## Conclusions

In this study, WRF-Hydro model was used to simulate the climate hydrological coupling situation of Daihai Basin, reproduce the climate change process and runoff temporal and spatial variation law from 1980 to 2020, and analyze the causes and future trend of hydrological elements change in Daihai Basin. The main conclusions are as follows: (1) the interannual variation of precipitation in Daihai Basin is sharp, with 401.75 mm as the average from 1980 to 1994; From 1995 to 2011, it fluctuated sharply; From 2012 to 2020, the fluctuation range is small. (2) the inflow of Daihai Lake presents a downward trend from 1980 to 2020; Since 2013, the runoff into the lake has tended to be flat. (3) Climate change and human activities are the decisive factors leading to the change of water quantity in Daihai Lake, among which human activities play a greater role. The correlation between cultivated land irrigation and industrial water use and the flow into the lake is very high, which has a great impact. (4) The protection measures of the Daihai Basin are very necessary now. If human activities are not controlled, the Daihai Lake will dry up in 2031–2033.

The climate hydrological coupling of long time series in arid area not only makes up for the lack of observation data, but also accurately obtains the long-term climate hydrological change information in the basin, which provides the basis for water resources management, disaster prevention and mitigation, and water ecological environment protection in arid area.
